# Toll-like receptor 4 signaling in liver injury and hepatic fibrogenesis

**DOI:** 10.1186/1755-1536-3-21

**Published:** 2010-10-21

**Authors:** Jinsheng Guo, Scott L Friedman

**Affiliations:** 1 Division of Digestive Diseases, Zhong Shan Hospital, Department of Internal Medicine, Shanghai Medical College, Fu Dan University, Shanghai, China; 2 Division of Liver Diseases, Mount Sinai Hospital, Mount Sinai School of Medicine, New York, NY, USA

## Abstract

Toll-like receptors (TLRs) are a family of transmembrane pattern recognition receptors (PRR) that play a key role in innate and adaptive immunity by recognizing structural components unique to bacteria, fungi and viruses. TLR4 is the most studied of the TLRs, and its primary exogenous ligand is lipopolysaccharide, a component of Gram-negative bacterial walls. In the absence of exogenous microbes, endogenous ligands including damage-associated molecular pattern molecules from damaged matrix and injured cells can also activate TLR4 signaling. In humans, single nucleotide polymorphisms of the *TLR4 *gene have an effect on its signal transduction and on associated risks of specific diseases, including cirrhosis. In liver, TLR4 is expressed by all parenchymal and non-parenchymal cell types, and contributes to tissue damage caused by a variety of etiologies. Intact TLR4 signaling was identified in hepatic stellate cells (HSCs), the major fibrogenic cell type in injured liver, and mediates key responses including an inflammatory phenotype, fibrogenesis and anti-apoptotic properties. Further clarification of the function and endogenous ligands of TLR4 signaling in HSCs and other liver cells could uncover novel mechanisms of fibrogenesis and facilitate the development of therapeutic strategies.

## Introduction

Toll-like receptors (TLRs) are evolutionarily conserved trans-membrane proteins originally identified in mammals on the basis of their homology with Toll, a *Drosophila *receptor that contributes to development in the embryo, and in the production of antimicrobial peptides against microorganism invasion in the adult fly [1,2].

TLRs are a family of pattern-recognition receptors that recognize pathogen-derived molecules termed pathogen-associated molecular patterns (PAMPs), which are structural components unique to bacteria, fungi and viruses. These ligands bind to TLRs, leading to signaling and activation of innate and adaptive inflammatory responses.

Ten TLRs have been identified in humans [3], which have individual or shared substrates for activation, and recognize microbes either on the cell surface or on lysosome/endosome membranes (Table 1). Toll-like receptor (TLR)4 was the first to be discovered, and is the most important Toll homolog; it responds primarily to its main ligand, lipopolysaccharide (LPS).

**Table 1:** Toll-like receptor (TLR) family and ligands

TLR	Ligands	Cellular location
TLR1	Triacylated bacterial lipopeptides	Cell membrane

TLR2	Triacylated bacterial lipopeptides	Cell membrane

TLR3	Double-stranded RNA produced by most viruses during replication	Endosomal compartment

TLR4	Lipopolysaccharide, low-molecular weight hyaluronic acid, heparin sulfate, saturated fatty acid, fibrinogen, fibronectin, heat shock proteins 60 and 70, high mobility group box-1, degraded matrix	Cell membrane

TLR5	Bacterial flagellin	Cell membrane

TLR6	TLR1 and 6 combine with TLR2 to distinguish the subtle differences between triacyl and diacyl lipopeptides	Cell membrane

TLR7	ssRNA viruses, influenza virus	Endosomal compartment

TLR8	ssRNA	Endosomal compartment

TLR9	Unmethylated CpG DNA found in bacteria, DNA virus	Endosomal compartment

TLR10	unknown	Cell membrane

^1^ssRNA = single-stranded RNA.

Recent studies indicate that TLR4 signaling can also be activated by some endogenous ligands from cellular compartments, which are released and/or increased during tissue injury and matrix degradation. These ligands are collectively referred to as damage-associated molecular patterns (DAMPs).

There is rapidly increasing knowledge both about TLR4 signaling in cells and about the association of single nucleotide polymorphisms (SNPs) of the TLR4 gene with the risks and mechanisms of human diseases. In liver, both parenchymal and non-parenchymal cell types express TLR4, which is actively involved in the response to injury from a variety of etiologies, including viral hepatitis, alcoholic and non-alcoholic liver diseases, autoimmune liver diseases and drug-induced liver diseases. TLR4 signaling is present in activated hepatic stellate cells (HSCs), the major fibrogenic cell type in injured liver, and mediates the inflammatory phenotype and survival of the cell. Recent studies have uncovered an important role for TLR4 signaling in liver fibrogenesis and the association of TLR4 polymorphisms with fibrosis risk.

In this review, we introduce TLR signal transduction and the functional role of TLR4 signaling in liver injury and fibrogenesis, pointing towards the potential to develop specific therapeutics.

## Components of the TLR4 signaling pathway

**Figure 1:**
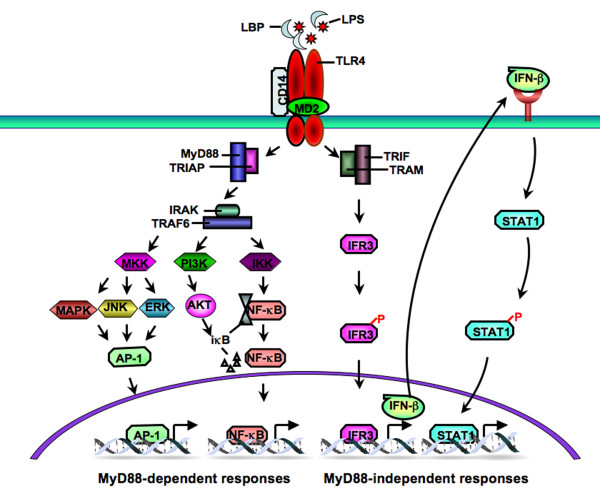
**Schematic overview of Toll-like receptor (TLR)4 signaling pathway**. LPS interacts with circulating LPS-binding protein (LBP) and binds to TLR4 on the cell membrane with two co-receptors (CD14 and myeloid differentiation protein (MD)2), activating myeloid differentiation factor (MyD)88-dependent and (MyD)88-independent TLR4 signaling via different adaptor proteins. The MyD88-dependent pathway signals through activation of iκB kinase (IKK) and mitogen activated protein kinase (MAPK) pathways, which in turn leads to activation of transcription factors nuclear factor (NF)-κB and activator protein (AP)-1, respectively, and controls the expression of pro-inflammatory cytokines and other immune related genes. In addition, phosphatidylinositol 3-kinase (PI3K) and AKT are also important factors downstream of MyD88 that mediate NF-κB activation. The MyD88-independent pathway is mediated by the TIR domain-containing adaptor inducing interferon-β (TRIF), which activates interferon regulatory (IRF)3 and induces the expression of interferon (IFN)-β and IFN-responsive genes

TLRs mediate a tightly integrated signal transduction cascade linking a series of protein-protein interactions with their ligands, receptors, co-receptors and adaptor proteins to convey downstream signals that control transcription [4] (Figure 1). Genes regulated by TLRs include cytokines and proteins controlling innate and adaptive immunity, cell survival and apoptosis, and fibrogenesis.

### TLR4 and co-receptors

Human TLRs are type I transmembrane glycoproteins that are structurally characterized by the presence of a leucine-rich repeat (LRR) domain in their extracellular structure, and a conserved Toll/interleukin (IL)-1 receptor (TIR) homology domain in their intracellular domains.

The extracellular domain is unique to each individual TLR, as it confers specificity for ligand recognition. For example, for the initiation of LPS to activate intracellular TLR4 signaling, the ligand first interacts with circulating LPS-binding protein (LBP) along with three LRR domain-containing proteins, TLR4 and the two co-receptors CD14 and myeloid differentiation protein (MD)2, together comprising the LPS receptor complex [5]. CD14 is a 55 kDa glycophosphatidylinositol-linked protein expressed on the surface of LPS-responsive cells such as macrophages and monocytes. Alone, CD14 cannot transduce a signal intracellularly because it lacks a transmembrane domain. Instead, it transfers LPS to a hydrophobic pocket within the MD2 glycoprotein, resulting in TLR4-dependent activation of cells. MD2 is a glycoprotein of approximately 17 to 25 kDa, and is present with TLR4 at the surface of various cell types, principally those of the myeloid and endothelial lineages. Despite the absence of a transmembrane domain, MD2 can attach to the cell surface via its interaction with TLR4 through specific epitopes. Human MD2 is an accessory molecule expressed on the cell surface that is not only required for cell-surface expression of TLR4, but also appears to be essential for the activation of the TLR4 signaling cascade. Activation of TLR4 by LPS absolutely requires the presence of the co-receptor MD2 for signaling, whereas some TLR4-mediated signals may still be generated in the absence of CD14.

Both CD14 and MD2 are soluble acute phase proteins [6]. They may act as a sink for LPS, and participate in opsonization and internalization of Gram-negative bacteria by human phagocytes.

### Adaptors

Upon LPS recognition, TLR4 undergoes oligomerization and recruits its downstream adaptors through interactions with the TIR domains. Four adaptor proteins (myeloid differentiation factor (MyD)88, MyD88-adaptor-like/TIR domain-containing adaptor protein (MAL/TIRAP) [7], TIR domain-containing adaptor inducing interferon-β (TRIF) and TRIF-related adaptor molecule (TRAM) [8,9]) transduce signals from all of the TIR domains and engage downstream signaling proteins. The function of a fifth adaptor, SARM (sterile alpha and HEAT/Armadillo motif protein), has yet to be defined. Different TLRs use different combinations of adaptor proteins to determine downstream signaling; TLR4 is the only known TLR that uses all these adaptor proteins.

TLR4 signaling has been divided into MyD88-dependent and MyD88-independent, TRIF-dependent pathways. The MyD88-dependent pathway signals through IL-1 receptor-associated kinase (IRAK)-1, IRAK-4, tumor necrosis factor (TNF) receptor-associated factor (TRAF)6, and transforming growth factor-β-activated kinase (TAK)1, which activates downstream iκB kinase (IKK) and mitogen-activated protein kinase (MAPK) pathways [10]. These events in turn lead to activation of the transcription factors nuclear factor (NF)-κB and activator protein (AP)-1, respectively, and control the expression of pro-inflammatory cytokines and other immune related genes. In addition, phosphatidylinositol 3-kinase (PI3K) and AKT are also important factors downstream of MyD88 that mediate NF-κB activation [11,12]. The MyD88-independent pathway is mediated by TRIF, which activates interferon regulatory factor (IRF)3 and induces the expression of interferon (IFN)-β and IFN-responsive genes [13]. The MyD88-independent pathway also mediates the late-phase activation of NF-κB and MAPK. The activation of two signaling pathways downstream of TLR4 is cell-specific and dependent on the dose of the ligands [14]. Interestingly, LPS is unable to activate the MyD88-independent pathway in terminally mature neutrophils [15].

### Transcription factors

At least three major transcriptional complexes are involved in TLR4 signaling: NF-κB, AP-1 and IFN regulatory factors (IRFs). These nuclear factors have important activities in HSCs (see below) and other resident liver cell populations. However, a comprehensive understanding of which transcriptional complexes are downstream of TLR4 in each cell type is lacking, and warrants further investigation.

### NF-κB

NF-κB is a pleiotropic protein complex that is activated from a sequestered, cytoplasmic form normally retained in the cytoplasm by binding to iκB (the NF-κB inhibitor protein) via pro-inflammatory extracellular signals and cellular stress. However, after iκB degradation, initiated by a complex signaling cascade initiated at the cell surface (for example, TLR4 signaling), the active form of NF-κB translocates into the nucleus, where it activates transcription.

NF-κB regulates hundreds of cellular genes including cytokines, chemokines, adhesion molecules that participate in the regulation of innate and adaptive immunity, and proteins that regulate cell-cycle progression (for example, cyclin D1) and cell survival (for example, Bcl-2, Bcl-xL and Bfl-1). Both pro- and anti-apoptotic gene products are regulated by NF-κB, depending on the cell type and the stimulus [16].

NF-κB activity can be perturbed in a variety of non-parenchymal and parenchymal liver cells during hepatic inflammation, fibrosis and the development of hepatocellular carcinoma, and regulates the interplay between immune, fibrogenic and oncogenic mediators [17]. Hepatic NF-κB is implicated in homeostatic processes such as: clearance of microbial pathogens, protection of hepatocytes from TNF-α-induced cell death, and compensatory proliferation of hepatocytes in response to loss of hepatic mass after liver injury [18]. NF-κB signaling has vast complexity, and the NF-κB activities are context-dependently regulated by subunit interactions, post-translational modification and recruitment of co-regulators.

Activated HSCs have persistently activated NF-κB, with a reduction in iκB expression. As a result, many NF-κB responsive genes are constitutively expressed in activated, but not in quiescent HSCs. Inhibiting NF-κB activation does not alter the activated cellular morphology of HSCs, or the expression of either α-smooth muscle actin (α-SMA) or collagen; however, NF-κB plays an important role in the anti-apoptotic property of activated HSCs and hepatic myofibroblasts [19]. It also mediates the regulation of the TGF-β1 pseudoreceptor BAMBI (bone morphogenic protein and active membrane bound inhibitor) in HSCs by TLR4-MyD88 activation, thus sensitizing the cell to TGF-β1 signaling [20].

### AP-1

The transcription factor AP-1 is composed of either homodimers or heterodimers of members of the Jun (c-Jun, v-Jun, Jun-B and Jun-D) and Fos (c-Fos, Fos-B, Fra-1 and Fra-2) families, and regulates cell proliferation, differentiation and survival. The regulation of AP-1 activity is complex, which can be achieved by modulating *jun *and *fos *gene transcription and mRNA turnover; Jun and Fos protein turnover; post-translational modifications of Jun and Fos proteins that modulate their transactivation potential; or interactions of AP-1 with other transcription factors that can either synergize or interfere with its activity. Various stimuli, such as physiological agents (growth factors, mitogens, polypeptide hormones, cell-matrix interactions and inflammatory cytokines), bacterial and viral infections, pharmacological compounds (phorbol esters), cellular stress (ultraviolet or ionizing radiation, hyperosmotic and heavy-metal stress), can induce AP-1 activity. These stimuli activate MAPK cascades by phosphorylating distinct substrates, mostly for p38, Jun amino-terminal kinase (JNK) and extracellular signal-regulated kinase (ERK), which enhance AP-1 activity.

In activated HSCs, AP-1 represents another family of transcription factors that has increased and persistent activity. AP-1 is a downstream effector of MAPK signaling that contributes to TGF-β1 and platelet-derived growth factor-induced HSC fibrogenesis and proliferation, respectively. In addition, AP-1 regulates tissue inhibitor of metalloproteinase (TIMP), matrix metalloproteinase (MMP) and other genes involved in matrix remodeling [21-23]. Jun D is the most important AP-1 factor in activated HSCs, as it is required for both TIMP-1 and IL-6 gene expression [22].

AP-1 can combine with several transcription factors to form complexes that synergistically mediate the transcription of fibrogenic genes. Examples of these crucial combinations include the cooperation of Smad3/4 complex with AP-1 in mediating TGF-β1-induced α2(I) collagen transcription [24], assembly of the Jun D and RunX factors at the TIMP-1 promoter to stimulate gene transcription [25], and NF-κB- and AP-1-mediated, IL-1β-stimulated TGF-β1 transcription [26].

### IRFs and STAT1

A family of IRFs regulate the transcription of IFN genes and IFN-stimulated genes. The MyD88-independent TRIF-dependent TLR4 pathway activates IRF3, and induces the expression of IFN-β and IFN-responsive genes [13,27], whereas IRF1 is activated via MyD88-dependent pathways. IRF1 rapidly translocates to the nucleus, and cooperates with IRF3 in response to LPS for the initial induction of target genes, including IL-27p28 [28].

Activation of signal transducers and activators of transcription 1(STAT1) by TLR4 signaling can occur directly via a protein kinase (PK)C-δ related mechanism [29] or indirectly by the induction of IFN-β via MyD88-independent IRF3 activation [13]. In the indirect mechanism, STAT1 is tyrosine-phosphorylated and dimerized after antocrine/paracrine interactions within IFN-β and IFNα/β receptors on the surface of cells, which result in the crossactivation of the receptor-associated Janus protein tyrosine kinases (JAKs). The activated STAT1 in turn regulates the expression of several STAT1-dependent genes [30], including genes involved in growth control and that mediate the responses of IFN types (for example, IFN-γ) to viral infections and other pathological agents.

### Downstream factors

The downstream factors regulated by TLR4 signaling include:

1. Effectors of the innate immune response: pro-inflammatory cytokines (TNF-α, IL-1, IL-6), chemotactic cytokines (monocyte chemotactic protein (MCP)-1, macrophage migration inhibitory factor (MIF)), pro-inflammatory proteins (inducible nitric oxide synthase (iNOS)), reactive oxygen species (ROS); adhesion molecules (intercellular adhesion molecule (ICAM)-1, vascular cell adhesion molecule (VCAM)-1) and other effectors of the innate immune response (for example, IFN-γ). Products of the inflammatory cascade such as IL-1, TNF-α and cyclooxygenase (COX)-2 can further amplify the inflammatory response.

2. Proteins that regulate cell-cycle progression (for example, cyclin D1) and the apoptotic threshold (for example, Bcl-2, Bcl-xL and Bfl-1).

3. The TGF-β1 pseudoreceptor BAMBI [20], which is downregulated by a TLR4-MyD88-NF-κB dependent pathway, thereby sensitizing HSCs to TGF-β1 signaling. Rregulation of BAMBI by TLR4 signaling provides a link between pro-inflammatory and profibrogenic signals [31].

### Negative regulation of TLR4 signaling

TLR4 signaling can be controlled at multiple levels by many regulators [4]. These (mostly inhibitory) pathways are necessary to protect the host from inflammation-induced damage. The key regulators include:

1. Radioprotective (RP)105, ST2L (also known as IL-1R1) and single immunoglobulin IL-1R-related molecule (SIGIRR), which are expressed on the cell surface, interact with TLR4, MD2, MyD88 and TIRAP, and inhibit the initiation of TLR4 signaling.

2. TRIAD3A (Triad domain-containing protein 3, variant A) and suppressor of cytokine signaling (SOCS)1, which are two E3 ubiquitin protein ligases involved in LPS/TLR4 signaling. TRIAD3A can interact with certain TIR domain-containing proteins such as TIRAP and TRIF, and promote their degradation. SOCS-1 was identified as a cytokine regulator that inhibits JAK-STAT signaling. SOCS-1 can induce the ubiquitination of TIRAP, leading to its subsequent degradation [32].

3. Other intracellular negative regulatory proteins act further downstream in the signaling pathway, and include IRAK-M (IRAK family member but lacks kinase activity), IRAK-2c and MyD88s (splicing variants of IRAK and MyD88), TRAF1 and TRAF4 (TRAF family members that interact with TRIF activity), A20 (a de-ubiquitinating enzyme that can remove ubiquitin moieties from TRAF6) [33], and syntenin [34].

4. Activating transcription factor (ATF)3, a member of the cAMP response element binding (CREB)/ATF family of transcription factors that negatively regulates TLR4-stimulated inflammatory responses by altering chromatin structure and interacting with regulatory regions of targeted genes (for example, NF-κB and AP-1 promoter binding sites) [35,36].

5. Let-7i, a cellular micro (mi)RNA, regulates TLR4 expression via post-transcriptional suppression [37]. The miRNAs are a newly identified class of endogenous small regulatory RNAs in the cytoplasm that associate with messenger RNAs based on complementarity between the miRNAs and the target mRNAs [38,39]. This binding causes either mRNA degradation or translational suppression, resulting in gene suppression at a post-transcriptional level. Human biliary epithelial cells (cholangiocytes) express let-7 family members, which are decreased in response to *Cryptosporidium parvum *infection and LPS, and are associated with upregulation of TLR4 and improved epithelial defense responses [37].

### Ligands

#### Exogenous ligands

The exogenous ligands of TLRs that are related to pathogen and host defense are referred to as PAMPs. LPS is the well-characterized PAMP for TLR4, and is the principal glycolipid component of the outer membrane of Gram-negative bacteria. TLR4 plays an important role in mediating LPS-induced inflammatory signaling and infectious diseases. TLR4 also recognizes proteins from respiratory syncytial virus, vesicular stomatitis virus and mouse mammary tumor virus [40-42].

#### Endogenous ligands

Besides its natural exogenous substrate LPS, there are endogenous substrates for TLR4 that also bind and activate TLR4 [43], including low molecular weight hyaluronic acid [44,45], free fatty acids (FFAs) [46], fibrinogen [47], fibronectin [48], heat shock proteins (HSPs) 60 and 70 [49,50] and high mobility group box (HMGB)1 [51]. *In vivo*, damage signals and intact extracellular matrix (ECM) degradation activate TLR4 [52].

Most of these endogenous ligands are released and/or increased during tissue injury and matrix degradation, and are now referred to as DAMPs. Release of DAMPs into the extracellular space is achieved by a number of mechanisms, including: leakage from necrotic cells; increased synthesis and post-translational modification in response to inflammation; and degradation of inactive precursors into TLR-mimetic degradation products in inflammatory environments. DAMPs mediate non-sterile inflammation by activating TLR4 signaling [53].

Most of the endogenous TLR4 ligands have been studied in macrophages, monocytes and neutrophils, and in cells from C3H/HeJ mice, which lack a functional TLR4 receptor because of a missense point mutation that results in the substitution of histidine for proline within the cytoplasmic portion of TLR4. The direct effect of these DAMPs on activated HSCs, which have intact TLR4 signaling [10,20,54], is yet to be delineated, and has potential importance for further clarifying the mechanisms of fibrogenesis.

##### HMGB1

HMGB1 is a highly conserved nuclear non-histone DNA-binding protein that functions as a structural co-factor that is crucial for proper transcriptional regulation in somatic cells. It induces bends in the helical DNA structure to facilitate multiple physical interactions of DNA with transcription factors, recombinases and steroid hormone receptors, and thus allows transcription and other nuclear events to take place. In addition to this transcription factor-like function, HMGB1 also has cytokine-like effects by promoting tumor metastasis and inflammation, which require its presence in the extracellular space. *In vitro *studies indicate that HMGB1 stimulates HSCs proliferation and expression of α-SMA [55].

Release of HMGB1 into the extracellular space is mediated by two mechanisms:

1. Acetylation of many of the lysine residues of HMGB1 that lie in proximity to its two nuclear-localization signals, thus reducing interaction with the nuclear importer protein complex and preventing nuclear re-entry while promoting secretion of HMGB1. This active HMGB1 secretion seems to occur predominantly in inflammatory cells.

2. Passive diffusion of HMGB1 from cells that undergo necrosis. The release of HMGB1 does not occur from apoptotic cells, presumably because HMGB1 is tightly bound to cruciform DNA and to hypoacetylated proteins within the nucleus of the apoptotic cell, whereas it is only loosely bound to DNA in necrotic cells. HMGB1 has been suggested as a signature DAMP that signals the presence of necrosis, and subsequently triggers inflammation.

HMGB1 is a late mediator of lethality, and contributes to the increased levels of circulating and tissue cytokines that are present hours to days after the initial exposure to LPS [56]. Transfection with dominant-negative constructs of TLR2 and TLR4 into macrophage cell lines demonstrates that both of these TLRs are involved in HMGB1-induced activation of NF-κB [51].

##### Mrp8 and Mrp14

The migration inhibitory factor-(MIF)-related protein (MRP)-8, encoded by Mrp8, also known as S100A8) and MRP14 (encoded by Mrp14, also known as S100A9) are the most abundant cytoplasmic proteins of neutrophils and monocytes. They belong to the calcium-binding S100 protein family and form a heterodimeric complex in a Ca^2+^-dependent manner. Both proteins are specifically released during the activation of phagocytes, and have an important role in the pathogenesis of sepsis.

Expression and release of MRP8-MRP14 complexes by phagocytes correlates with disease activity in many inflammatory disorders. The complexes induce an inflammatory and prothrombotic response in endothelial cells *in vitro*, and promote leukocyte-endothelial cell interactions [57,58]. The Mrp8-Mrp14 complexes amplify the endotoxin-triggered inflammatory responses of phagocytes, enhance the expression of TNF-α, and promote lethality during septic shock [59]. Mrp14-deficient mice have decreased systemic inflammation, lower cytokine plasma concentrations, and less severe liver damage during abdominal sepsis [60]. Using phagocytes obtained from mice expressing a non-functional TLR4 mutant protein, and HEK293 cells (human embryonic kidney cells) replaced with exogenous expression of TLR4, MRP8 was demonstrated to interact specifically with the TLR-MD2 complex, thus representing an endogenous ligand of TLR4 [59].

##### Fibrinogen

Fibrinogen is a 340 kDa multimeric glycoprotein that has crucial functions in vascular hemostasis. It is normally confined to the vasculature, but at sites of inflammation, increased vascular permeability allows plasma extravasation. The concentration of circulating fibrinogen and the deposition of local fibrinogen increases significantly during inflammatory responses. LPS and fibrinogen stimulate the expression of similar cytokines (for example, IL-6) and chemokines (for example, MCP-1), and activate the transcriptional factors NF-κB and AP-1 in macrophages, fibroblasts and endothelial cells [47,61]. Macrophages from C3H/HeJ mice that express mutant TLR4 fail to respond to fibrinogen, indicating that innate responses to fibrinogen and bacterial endotoxin may converge at the evolutionarily conserved Toll-like recognition molecules [47].

##### Fibronectin

Cellular fibronectin, which contains alternatively spliced exons encoding type III repeat extra domain (ED)A and EDB, are produced in response to tissue injury. Fragments of fibronectin or specific fibronectin domains are believed to play important roles in physiological and pathological processes, including tissue remodeling in response to inflammation. The responses of cells exposed to recombinant EDA or EDA-containing fibronectin are similar to those observed when cells are treated with bacterial LPS, including the induction of genes encoding proinflammatory cytokines and MMPs. Recombinant EDA, but not other recombinant fibronectin domains, activates human TLR4 expressed in a cell type (HEK293) that normally lacks this TLR. EDA stimulation of TLR4 is dependent upon co-expression of MD2, a TLR4 accessory protein [48].

##### Hyaluronan

Hyaluronan is a negatively charged, high molecular weight glycosaminoglycan, which is ubiquitously distributed in the ECM and is a component of the basement membrane. At sites of inflammation and tissue destruction, high molecular weight hyaluronan can be broken down to lower molecular weight hyaluronan fragments via oxygen radicals and enzymatic degradation. In contrast to high molecular weight hyaluronan, low molecular weight hyaluronan has cytokine-like properties, and induces inflammatory gene expression in epithelial cells, endothelial cells, fibroblasts, dendritic cells and macrophages. These effects are at least partially TLR4-dependent, as shown in studies using a TLR4 blocking antibody and TLR4-deficient mice [44,45]. Because the disruption of basement membranes is typically associated with injury, recognition of low molecular weight hyaluronan by TLR4 and other receptors is part of an injury recognition system.

##### Heat shock proteins

HSPs are remarkably conserved proteins in all living organisms. Their expression is induced in response to a variety of physiological and environmental insults. In the cytosol, these proteins play an essential role as molecular chaperones by assisting the correct folding of nascent and stress-accumulated misfolded proteins, preventing protein aggregation, facilitating transport of proteins, and supporting antigen processing and presentation. Following stress, intracellular HSPs fulfill protective functions and thus prevent lethal damage. By contrast, membrane-bound or extracellular HSPs act as danger signals and elicit immune responses mediated by either the adaptive or innate immune system.

HSPs 60 and 70 are two HSPs that elicit potent inflammatory responses in cells of the innate immune system, which are dependent on functional TLR4. Mouse or human macrophages and endothelial or smooth muscle cells elicit a pro-inflammatory response when incubated with recombinant human HSP60. The response includes the upregulation of adhesion molecule expression and the release of inflammatory mediators such as IL-6 and TNF-α, as well as IL-12 and IL-15, two cytokines that are essential in driving the T helper (Th)1 response. Macrophages of C3H/HeJ mice carrying a mutant TLR4 are unresponsive to HSP60. Similarly, HSP70 can induce TNF-α production by human monocytes, which is inhibited by anti-TLR4 [49]. These findings suggest that HSP60 and HSP70 are endogenous ligands of the TLR4 complex, and that there is a role for TLRs in innate immune discrimination of normal versus stressed or damaged tissue cells.

##### Saturated fatty acids

Lipid A, which possesses most of the biological activities of LPS, is acylated with saturated fatty acids (SFAs). Removal of these acylated SFAs from lipid A not only results in complete loss of endotoxic activity, but also makes the deacylated lipid A act as an antagonist to native lipid A, suggesting that the FAs that are acylated in lipid A play a crucial role in ligand recognition and receptor activation for TLR4.

Lee *et al*. [11,46] showed that an SFA (lauric acid), but not unsaturated FAs, could induce NF-κB activation and COX-2 expression. This effect is mediated through the TLR4-PI3K-AKT signaling pathway, as the induction of inflammatory markers was also inhibited by a dominant-negative mutant of TLR4, MyD88, IRAK-1, TRAF6 or IκBα in macrophages (RAW264.7) and 293T cells transfected with TLR4 and MD2, and the NF-κB activation was inhibited by the AKT inhibitor LY294002, and by dominant-negative PI3K or AKT. Lauric acid also upregulates the expression of co-stimulatory molecules (CD40, CD80 and CD86), major histocompatibility complex (MHC) class II molecules, and cytokines (IL-12p70 and IL-6) in bone marrow (BM)-derived dendritic cells (DCs). The dominant-negative mutant of TLR4 or its downstream signaling components can inhibit lauric acid-induced expression of a CD86 promoter reporter gene. By contrast, an n-3 polyunsaturated FA (docosahexaenoic acid), inhibits TLR4 agonist (LPS)-induced upregulation of the co-stimulatory molecules, MHC class II molecules, and cytokine production. Similarly, DCs treated with lauric acid show increased T-cell activation capacity, whereas docosahexaenoic acid inhibits T-cell activation induced by LPS-treated DCs [62]. Studies using a co-culture system of adipocytes and macrophages (C3H/HeN and C3H/HeJ peritoneal macrophages, RAW264 macrophages) showed that SFA released from hypertrophied adipocytes via the macrophage-induced adipocyte lipolysis serve as a naturally occurring ligand for TLR4, thereby inducing the inflammatory changes in both adipocytes and macrophages through NF-κB activation [63,64].

These results imply that TLR4 is involved in sterile inflammation and immune responses induced by non-microbial endogenous molecules. These findings shed new light on how different types of dietary FAs differentially modulate immune responses that could alter the risk of many chronic diseases [11,46,62-64]. However, controversy exists about whether FA-induced TLR4 activation might be due to artifacts such as contamination by LPS of the FA preparations [65]. In addition, several lipoproteins such as low-density lipoprotein might be shuttle molecules for LPS; thus, it is possible that TLR4 activation in the *in vivo *models of obesity is stimulated by LPS bound to FFAs.

## TLR4 signaling in liver cells

Compared with other organs, healthy liver contains low mRNA levels of TLRs and signaling molecules such as MD2 and MyD88, which may account for the high tolerance of the liver to TLR ligands from the intestinal microbiota, to which the organ is constantly exposed. Damaged liver has increased expression of TLR4 and its co-receptors, which sensitize the inflammatory cascade mediated by TLR4 signaling in the injured organ [66]. In liver, TLR4 is expressed by both the hepatocytes and non-parenchymal cells (NPCs), including liver sinusoidal endothelial cells (LSECs) and Kupffer cells (KCs). NPCs display a cell type-specific activation profile in response to the stimulation by TLR ligands [67].

### Hepatocytes

Hepatocytes fulfill metabolic and detoxifying functions in the liver, and are important mediators of the acute phase response. Hepatocytes express TLR4, and respond to LPS by inducing serum amyloid A, cytochrome P450, superoxide dismutase activity, adhesion molecule, TNF-α, IL-6 and LBP. However, this response is fairly weak, with only twofold elevated levels of serum amyloid A and a less than twofold induction of most upregulated genes in a microarray after administration of LPS. Moreover, LPS doses of 100 ng/ml and higher are required to elicit significant effects in hepatocytes.

Hepatocytes play a major role in the uptake of LPS and its removal from the systemic circulation, by secreting LPS into the bile. The uptake of LPS by hepatocytes *in vivo *is through a CD14-TLR4-MD2-dependent mechanism, and is mediated by β2-integrin-induced p38 MAPK activation [32,68]. Endogenous TLR4 ligands such as extracellular HSP72, a strongly stress-inducible 72-kDa protein that is released during ischemia-reperfusion injury (IRI), can stimulate hepatocytes to produce MIP-2, IL-6 and TNF-α via TLR4-NF-κB-dependent signaling [69]. Studies of KC depletion in transgenic mice expressing the hepatocyte-specific hepatitis C virus (HCV) nonstructural protein NS5A suggest that hepatocytes can be the primary cellular site of both TLR4 upregulation and its pathologic consequences in HCV infection [70].

### Kupffer cells (KCs)

KCs are the resident macrophages of the liver. They play a crucial role in host defense, which is linked to the ability of these cells to phagocytose, process and present antigen. KCs also secrete various pro-inflammatory mediators including cytokines, prostanoids, nitric oxide and reactive oxygen intermediates.

KCs are among the first cells in the liver to be exposed to gut-derived toxins such as LPS, and they orchestrate the inflammatory responses within the liver. KCs express TLR4 and respond to LPS by producing pro-inflammatory cytokines (for example, TNF-α, IL-1β, IL-6) and ROS (for example, superoxide and nitric oxide) [67]. Notably, KCs mediate the majority of cytokine and chemokine expression in liver after LPS injection. LPS stimulates TLR4 on KCs to enhance hepatocyte damage, increase leukocyte infiltration and secrete pro-fibrogenic cytokines. Moreover, KCs stimulated by TLR1, 2, 4 and 6 can activate allogenic T cells. By contrast, freshly isolated human KCs secrete the anti-inflammatory cytokine IL-10 in response to stimulation with LPS, which contributes to the downregulation of pro-inflammatory cytokines. Thus, KCs may have a higher LPS tolerance to adapt to the special circumstances in their anatomical location, in which they frequently encounter low levels of LPS even under normal conditions.

### Hepatic stellate cells (HSCs)

HSCs are the predominant ECM-producing cell type in the liver. Apart from the important fibrogenic activity of HSCs, the cells have emerged as key effectors of the liver's inflammatory response, rather than being simply targets of inflammation.

Another important property of activated HSCs is their resistance to pro-apoptotic stimuli. Induction of HSC apoptosis has been proposed as a strategy to treat liver fibrosis. Activated human HSCs express LPS-recognizing receptors such as CD14, TLR4 and MD2, and they have intact TLR4 signaling. Activated HSCs respond to even low concentrations of LPS with the activation of IKK-NF-κB and JNK, secretion of pro-inflammatory cytokines (IL-6, IL-8 and TNF-α), chemokines (MCP-1, MIP-2, ICAM-1, RANTES (regulated upon activation, normal T cell expressed and secreted) and C-C chemokine receptor (CCR)5), and expression of adhesion molecules [10,71]. In addition, an anti-apoptotic effect of TLR4 signaling has been reported in macrophages, cancer cells and HSCs. NF-κB, MAPK and PI3K/Akt signaling and downstream cytokines (for example, IL-6) and anti-apoptotic proteins (for example, Bcl-2) elicited by TLR4 activation play an active role in HSC survival [72]. Moreover, TLR4 signaling in HSCs may be more important than in KCs in mediating fibrogenesis, in part by downregulation of the inhibitory TGF-β1 pseudoreceptor, BAMBI [20,31]. Chimeric mice with TLR4 mutant KCs show the same degree of hepatic inflammation and fibrosis as their wild-type counterparts, whereas mice with TLR4 mutant HSCs, but wild-type TLR4 KCs, show a similar resistance to experimental fibrosis as do TLR4 mutant C3H/HeJ mice, indicating the crucial role of TLR4 expression on HSCs. HSCs, in addition to KCs, may be a target for LPS-induced liver injury, and provide a direct link between inflammatory and fibrotic liver injury. The direct regulation of HSC gene expression by LPS represents a novel mechanism for hepatic injury and fibrosis.

### Liver sinusoidal endothelial cells (LSECs)

Similar to liver macrophages, LSECs are highly responsive to acute endotoxemia, with induction of iNOS, COX-2, IL-1β, TNF-α and 5-lipoxygenase genes. This activity is largely dependent on TLR4 [73]. In culture, LSECs respond to ligands of TLR1, 2, 3, 4, 6, 8 and 9 by producing TNF-α, to ligands of TLR3 and 4 by producing IL-6, and to TLR3 ligands by producing IFN-β [67]. LPS decreases vitamin K-dependent protein (P)S expression in hepatocytes and LSECs, which is mediated by MEK-ERK signaling and NF-κB activation, with the involvement of membrane-bound CD14 and TLR4 [74].

### Biliary epithelial cells

The biliary tract directly communicates with the intestinal tract, and is therefore directly exposed to microorganisms from the gut. Human cholangiocytes or (biliary epithelial cells; BECs) express all 10 known TLRs, and activation of TLRs triggers an array of epithelial defense responses, including production and release of cytokines or chemokines (for example, TNF-α, MCP-1, IL-6 and IL-8) and anti-microbial peptides [75-77]. TLR2 and TLR4 signaling mediate cholangiocyte responses, including production of human β-defensin 2 against *C. parvum *via TLR-associated activation of NF-κB. Human cholangiocytes express members of the let-7 miRNA family, at least one of which, let-7i, directly regulates TLR4 expression via a MyD88-NF-κB-dependent mechanism. Following microbial insult, cholangiocytes decrease let-7i expression and consequently upregulate TLR4 expression via translational suppression in infected cells, which contributes to epithelial immune responses to microbial infection [37]. Endotoxin tolerance is present within the intrahepatic biliary tree, which is important in maintaining innate immune biliary homeostasis. The tolerance is possibly induced by the expression of IRAK-M in the intrahepatic biliary epithelium [78].

### Hepatic dendritic cells (DCs)

DCs are classic antigen-presenting cells that present peripheral antigens to T cells in lymph nodes, regulate T cell differentiation (tolerance or immunity, Th1 or Th2 polarization) and initiate specific immune responses. Their maturation is vital for the induction of antigen-specific T-lymphocyte responses. DCs express TLRs, and as a result of TLR triggering, DCs upregulate co-stimulatory molecules, secrete immunomodulatory cytokines such as IL-12, and increase antigen processing and presentation to B and T lymphocytes. Thus, TLRs function to alert the immune system to infection by stimulating DCs, which act as a bridge between the innate and adaptive immune systems.

Liver DCs exhibit a comparatively high threshold for stimulation by LPS, which may be explained by their low expression of TLR4. This unresponsiveness can be at least partially overcome by high LPS levels that exceed those encountered in the absence of clinical infection [79]. LPS-mediated TLR4 signaling leads to maturation of DCs [80]. Hepatic DCs respond to TLR1, 2, 4 and 9 ligands by both upregulation of CD40 and activation of allogeneic T cells. TLR3 and TLR4 stimulation in DCs induces co-stimulatory molecules and cytokines [67]. In a mouse model of diabetes, HMGB1 upregulates CD40 expression and enhances IL-12 production by DCs, leading to natural killer (NK)T cell activation and subsequent NKT cell-dependent augmentation of IFN-γ production, with the early loss of transplanted islets [81]. AM3, a mixture containing immunoregulatory glycoconjugates, induces functional maturation of monocyte-derived DCs from patients with chronic HCV infection and healthy donors, and stimulates the secretion of molecules with antiviral properties in a TLR4-dependent manner [82].

### Lymphocytes

Lymphocytes constitute 25% of the non-parenchymal resident cells in the normal human liver, and the sub-populations differ numerically according to normal conditions or the existence of stresses such as inflammation and steatosis [83]. TLRs are widely expressed by immune cells, including T and B lymphocytes. Conventional CD4+ T helper cells, cytotoxic T lymphocytes (CTL) and naturally arising regulatory T cells (Tregs) all express TLRs. TLR triggering on innate immune cells results in the induction of pro-inflammatory cytokines, phagocytosis and subsequent innate effector mechanisms, including an oxidative burst [84].

CD4+ CD25+ Foxp3+ Tregs, a key player in maintaining peripheral T cell tolerance [85], express TLR4 and other TLRs. Direct engagement of TLR4 by LPS on Tregs upregulates several activation markers, and induces Treg proliferation or survival without the need for TCR ligation. More importantly, LPS-treated Tregs exert enhanced function *in vitro *and remain suppressive *in vivo *[86]. Regulation of Treg function via TLRs constitutes an important immunosuppressive cellular mechanism to curtail TLR hyperactivity, thereby avoiding sepsis and autoimmune diseases [87].

## TLR4 signaling in liver injury

TLR4 signaling is a 'good' response in promoting pathogen eradication and initiating liver regeneration. It is essential in the generation of both innate and adaptive immune responses against pathogens (for example, salmonella) [88]. TLR4-deficient C3H/HeJ mice have an increased microbial burden and mortality after infection [88].

**Figure 2:**
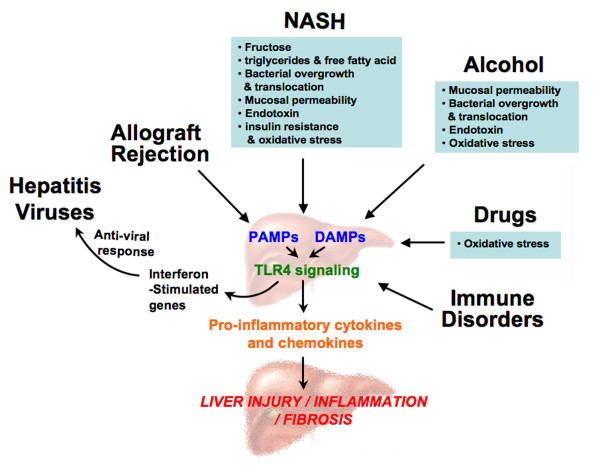
**Activation of the Toll-like receptor (TLR)4 signaling in liver injuries**. Exogenous and endogenous ligand(s) of TLR4 are increased during liver injuries caused by various etiologies such as nonalcoholic steatohepatitis (NASH). Activation of TLR4 by both pathogen-associated molecular patterns (PAMPs; exogenous ligands, for example, lipopolysaccharide (LPS)) and damage-associated molecular patterns (DAMPs; endogenous ligands, for example, high mobility group box (HMGB)1) induces pre-inflammatory cytokines and chemokines, thereby enhancing liver inflammation, injury and fibrogenesis. TLR4 activation also enhances interferon (IFN)-β and IFN-stimulated gene expression, which have inhibitory effects on hepatitis viruses.

However, TLR4 also plays a deleterious role in hepatic inflammation and injury arising from many causes (Figure 2). TLR activation induces pro-inflammatory cytokine cascades, which contribute to the pathophysiology and clinical outcome of severe liver injuries. Genetic deletion or mutation of TLR4 reduces macrophage infiltration and liver injuries in animals with experimentally induced liver damage.

### Drug-induced liver diseases

TLR4 is involved in the generation of steatosis, congestion and necrosis from paracetamol (acetaminophen; APAP) [89] through release of inflammatory cytokines (TNF-α), induction of iNOS and peroxynitrite, and depletion of glutathione. These events occur in response to LPS and possibly endogenous ligands released from the ECM during chemical or mechanical injury. Such injuries can further amplify systemic inflammatory immune responses by enhancing TLR4 reactivity, and can also result in leukocyte sequestration along with increased pro-inflammatory cytokine and chemokine levels. Serum hyaluronic acid is elevated in APAP-mediated liver injury in humans, and has been proposed as a prognostic indicator of survivability after APAP overdose [90,91].

### Viral hepatitis

Components of hepatitis viruses are ligands for TLR3, TLR7, TLR8 and TLR9, but not for TLR4. However, innate immune responses induced by TLR4 signaling may antagonize anti-hepatitis B virus (HBV) infection *in vivo *through the induction of IFN-α/β, iNOS and HBV-specific immune responses.

Several TLRs, including TLR4, block HBV replication through their ability to upregulate IFNs [92]. Similarly, *in vitro *studies indicate that activation of TLR3 and TLR4 by their ligands in non-parenchymal liver cells (in particular KCs and LSECs) is able to induce IFN-β- and IFN-stimulated genes, which leads to potent suppression of HCV replication [67,93]. TLR4 is upregulated in the hepatocytes of patients with chronic HBV, indicating a potentially important interaction. TLR4 and TLR6 are downregulated in HBV-infected peripheral blood monocytes, and these cells also have a decreased cytokine response to TLR2 and TLR4 ligands. By contrast, TLR4 expression is increased in the peripheral blood monocytes of patients with chronic HCV, along with increased cytokine production, including that of IFN-β, TNF-α, IL-6 and IL-8. The number of Tregs is significantly higher in these patients, which correlates with HCV genotype and viral load [94,95]. The HCV non-structural protein (NS)5A and alcohol synergistically induce hepatocellular damage and transformation via amplified and/or sustained activation of TLR4 signaling, with the induction of Nanog downstream of TLR4 signaling. Induction of this stem cell marker may contribute to HCV-induced liver oncogenesis enhanced by alcohol [70].

### Non-alcoholic fatty liver diseases

Obesity, insulin resistance (IR) and oxidative stress are major pathogenetic determinants of non-alcoholic fatty liver disease (NAFLD). Insulin receptor-mediated tyrosine phosphorylation of insulin receptor substrates (IRS) leads to the activation of downstream pathways (PI3K/AKT and MAPK) responsible for insulin action on glucose uptake and suppression of gluconeogenesis, cell growth and differentiation. Inflammatory cytokines such as TNF-α and IL-1 interfere with insulin signaling by provoking IRS serine phosphorylation and thus inactivate its activity in insulin signaling, causing IR [96]. Production of IL-6 or TNF-α also blunts insulin signaling in hepatocytes and muscle by increasing suppressor of cytokine signaling (SOCS)1, SOCS3 and nitric oxide (NO).

There is a vicious circle of aggravating IR based on hepatic steatosis and inflammation. IR leads to increased circulating FFA concentration and ectopic fat accumulation, which impede insulin-mediated glucose uptake in skeletal muscle and elevate glucose production in liver. By contrast, TLR4 on adipocytes and macrophages is a sensor of elevated FFA concentrations, which initiates inflammatory and thus insulin-desensitizing processes, leading to the development of NAFLD. Fatty acids, in particular SFAs, utilize TLR4 to induce NF-κB activation and pro-inflammatory cytokine expression in macrophages, adipocytes, vascular endothelial cells and liver.

TLR4 is a molecular link between nutrition, lipids and inflammation [97]. Triglycerides potentiate the inflammatory response of KCs to LPS in producing inducible NOS, TNF-α, IL-1β, IL-6 and granulocyte colony-stimulating factor *in vitro *[98]. Dietary fructose intake is also associated with NAFLD. Intake of high levels of fructose results in high triglyceride levels in plasma and their deposition in liver, as well as intestinal bacterial overgrowth and increased intestinal permeability, leading to elevated endotoxins and activation of TLR4 signaling [99,100].

### Alcoholic steatohepatitis

In alcoholic liver disease, endotoxemia may play a primary role in the induction of liver damage as a consequence of activating TLR4 signaling, thereby initiating an inflammatory cascade in liver cells [101]. Ethanol increases the circulating levels of gut-derived endotoxin, as a result of alteration of gut permeability, modification of the gut flora and changes in the rates of endotoxin clearance [102]. Alcohol induces LBP and TLR4, and increases responsiveness to gut-derived endotoxin. Binding of LPS to CD14/TLR4 on KCs activates production of cytokines and oxidants, primary mediators of early ethanol-induced liver injury. Furthermore, cytokine and oxidant production lead to T cell recruitment, HSC activation and collagen production in the liver of patients with alcoholic steatohepatitis [103]. Antibiotics and lactobacilli reduce liver injury in animals chronically fed alcohol.

### Autoimmune liver diseases

In primary biliary cirrhosis, TLR4 is expressed in bile duct epithelial cells and periportal hepatocytes, and may be involved in the inflammation and tissue destructive process of bile ducts and the interface hepatitis of primary biliary cirrhosis (PBC) [104]. These findings indicate that bacterial pathogens and TLR4 may contribute to the inflammatory response in PBC.

Stimulation of BECs with primary sclerosing cholangitis IgG, but not control IgG, induces expression of TLR4 and TLR9, and specific phosphorylation of both ERK, and the transcription factors ELK-1 and NF-κB. In BECs, a specific inhibitor of ERK1/2 abrogates phosphorylation of ELK-1 and protein expression of TLR4 but not TLR9 [105].

### Ischemia-reperfusion injury (IRI)

IRI occurs in solid organ transplantation, hemorrhagic shock, diverse surgical procedures, and heart failure. It is defined as an ischemic insult that induces delayed dysfunction and damage due to activation of inflammatory pathways, in which the TRL4 signaling pathway appears to be crucial. IRI induces a biphasic pattern of liver injury, with an acute phase occurring within the first 6 hours of reperfusion, characterized by a burst of pro-inflammatory cytokines and formation of ROS. This is followed by a late phase from 6 to 24 hours after ischemia, characterized by sequestration of neutrophils into the liver.

IRI is a tightly coordinated and sequential process mediated by the integration of both innate and adaptive immune reactions. TLR4 signaling by non-parenchymal cells is required for initiation of hepatic IRI [106,107]. Analysis of TLR4 chimeric mice with hepatic IRI indicates that mutation of TLR4 within either BM-derived or non-BM-derived TLR4 reduces hepatic IRI in the late reperfusion stage via reduced cytokine release and neutrophil infiltration, whereas non-BM-derived TLR4 regulates the expression of ICAM-1 on hepatocytes and LSECs, exacerbating the injury [108]. TLR4 signaling is also a putative repressor of heme oxygenase-1, which is cytoprotective and antioxidative in hepatic IRI [109].

### Allograft rejection after liver transplantation

Although continuous improvements in immunosuppression and clinical management have contributed to increased graft survival, acute rejection remains common after liver transplantation (25 to 49% of cases). The innate immune response, which is important in regulating the quality of the adaptive immune response, plays a prominent role in immune recognition of solid organ allografts. The family of TLRs is expressed on a variety of cell types, including antigen-presenting, epithelial and endothelial cells.

TLRs are a crucial link in activating dendritic cell maturation programs that induce adaptive immune responses. TLRs may be activated by some endogenous agonists, thereby participating in allograft responses. Significantly higher TLR4 and TLR2 expression is present on circulating monocytes in recipients of liver transplantation with acute rejection, compared with those who are clinically stable with normal liver function. Thus, elevated TLR2 and TLR4 may be candidates for early prediction of acute rejection after liver transplantation [110].

There are differential effects of donor and recipient TLR4 signaling in human liver transplantation. Donor TLR4 contributes to sterile injury after cold preservation, whereas the recipient TLR4 genotype is linked to poor allograft survival among HCV-infected recipients [111]. Myeloid dendritic cells (MDCs) of donor origin detached from liver grafts that migrate into the recipient express higher levels of TLR4 than do blood or splenic MDCs. These MDCs are sensitive to stimulation with physiological concentrations of LPS, produce pro-inflammatory and anti-inflammatory cytokines, and are capable of stimulating allogeneic Th1 responses. Thus, MDCs may contribute to liver graft rejection rather than tolerance [112].

### Cirrhosis

TLR4 regulation is altered in monocytes from patients with cirrhosis. Under normal conditions, monocytes release pro-inflammatory mediators such as TNF-α, IL-1 and IL-6 in response to LPS stimulation, which promote systemic inflammatory reactions including fever and leukocytosis. Patients with cirrhosis have chronic endotoxinemia, with elevated serum levels of TNF-α, IL-1β and IL-6 due to activated KCs and decreased hepatic clearance. Patients with cirrhosis also lack both LPS-mediated upregulation of pro-inflammatory cytokines by peripheral blood mononuclear cells and systemic reactions such as fever and leukocytosis, whereas bacterial infections are extraordinarily frequent. Patients with cirrhosis also have a higher basal level of TLR4 expression, which is induced upon LPS stimulation and is persistently upregulated after 24 h of incubation with LPS [113].

## TLR4 signaling and liver fibrogenesis

### Important link between TLR4 signaling and enhanced fibrogenesis

Fibrosis is characterized by an excessive deposition of ECM protein, impairing normal liver function, and ultimately leading to cirrhosis and organ failure. Inflammation and tissue injury are important factors that initiate and promote liver fibrosis [114-116]. Chronic inflammation and fibrogenesis are a dynamic aggregate of lymphocytes, macrophages and stromal cells linked by autocrine and paracrine interactions [117]. Inflammatory cells belonging to the innate (for example, NK cells and macrophages) and adaptive immune response (T and B cells) participate in liver injury and fibrogenesis. TLR signaling in the course of liver injury by hepatitis viruses and other etiologies contributes significantly to the activation and interaction of inflammatory cells, myofibroblasts and the matrix microenvironment.

**Figure 3:**
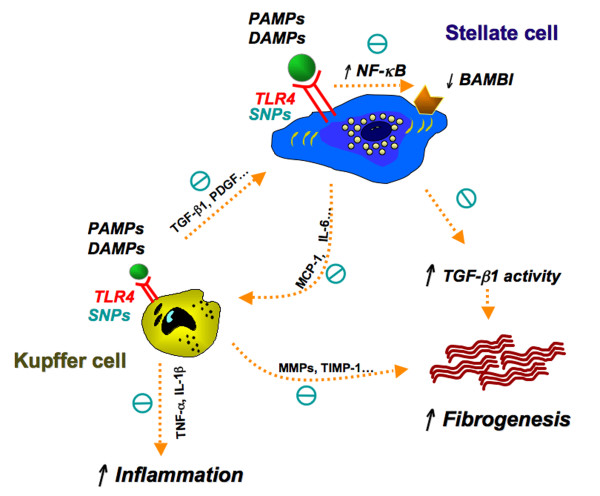
**Hypothesis: fibrogenic signaling induced by Toll-like receptor (TLR)4 in hepatic stellate cells (HSCs) and Kupffer cells (KCs), and the effect of TLR4 single nucleotide polymorphisms (SNPs)**. HSCs have intact TLR4 signaling. Both pathogen-associated molecular patterns (PAMPs; exogenous ligands, for example, LPS) and damage-associated molecular patterns (DAMPs; endogenous ligands, for example, HMGB1) may activate TLR4 in HSCs, signaling through NF-κB, leading to downregulation of the transmembrane inhibitory TGF-β1 pseudoreceptor bone morphogenic protein and active membrane bound inhibitor (BAMBI) on HSCs, a key fibrogenic cell in liver. As a result, net TGF-β1 activity increases, enhancing extracellular matrix production. Concurrently, TLR4 signaling in HSCs also increases the activity and migration of KCs. Activation of TLR4 signaling on KCs enhances the interaction within KCs and HSCs by activating the production of profibrogenic factors. Polymorphisms of the TLR4 gene, which decrease the recognition of PAMPs and DAMPs by TLR4 on HSCs and KCs, decrease the activation of TLR4 signaling and downstream production of proinflammatory cytokines, chemokines and fibrogenic proteins such as BAMBI. The TLR4 single nucleotide polymorphisms (SNPs) confer an overall protective effect against inflammation and fibrogenesis.

TLR4 signaling is activated in acute infection to clear the pathogen, but contributes to liver scarring in chronic disease. TLR4 and its ligands mediate their effects in liver fibrosis through different mechanisms. First, TLR4 downregulates the TGF-β1 pseudoreceptor BAMBI to sensitize HSCs to TGF-β1-induced signals [20]. Second, TLR4 activation also upregulates cytokine and chemokine secretion from cells with inflammatory phenotypes such as KCs and HSCs [10,72,88,118] (Figure 3). HSCs act as important effectors of the liver's inflammatory response by regulating leukocyte trafficking and KC recruitment and activation via secretion of cytokines and chemokines [119,120].

TLR4 was identified as one of seven genes associated with increased risk of developing cirrhosis in patients with chronic hepatitis C [72,121]. LPS and endogenous TLR4 ligands are increased in the serum and livers of patients with liver cirrhosis and of animals with experimental chronic liver disease. CD14- or LBP-deficient mice are resistant to the liver injury and fibrosis induced by bile duct ligation [122]. Studies using TLR4 mutant C3H/HeJ mice have demonstrated that hepatic inflammation and fibrosis are strongly decreased in the TLR4 mutant C3H/HeJ strain after bile duct ligation or carbon tetrachloride administration [20]. The cellular mechanisms underlying the fibrosis-promoting effects of TLR4 in the liver are not yet elucidated.

### TLR4 polymorphisms and the functional consequences on liver fibrosis

SNPs of TLRs influence the vigor of immune responses in bacterial, viral and parasitic infections. More than 100 SNPs have been identified in human TLR4 genes, of which the TLR4 T399I and D299G are two common (frequency 0 to 20% across different ethnicities), highly co-segregated (80% co-segregation rate), non-synonymous polymorphisms within the extracellular domain of the TLR4 protein. These SNPs may affect the strength of interactions with either agonist(s) and/or co-receptors, leading to decreased recognition of ligands in an agonist-independent manner [123-125]. These TLR4 SNPs are primarily associated with a blunted response to inhaled LPS in humans [126]. Many studies report the association between these TLR4 genetic polymorphisms and disease risk. They are associated with susceptibility to infectious diseases including Gram-negative bacterial infection [127,128], severe malaria [129], bronchitis [130-132], and diseases as disparate as inflammatory bowel disease [130], early onset pre-eclampsia [133], *Helicobacter pylori *infection, and gastric cancer [134]. TLR4 SNPs reduce the risk of early acute allograft rejection. They are not associated with the risk or severity of either rheumatoid arthritis or systemic lupus erythematosus [135-138]; multiple sclerosis [139,140]. spondylarthropathies [141], cerebral ischemia [142], juvenile idiopathic arthritis [143]; and outcome of angiography [144].

In contrast to the reduced risk of early acute allograft rejection [111,145], TLR4 SNPs are associated with delayed progression of hepatic fibrosis [121,146]. A gene-centric functional genome scan in patients with chronic hepatitis C yielded a Cirrhosis Risk Score (CRS) signature consisting of seven SNPs that may predict the risk of developing cirrhosis [121]. Of these seven SNPs, the major CC allele of TLR4 encoding p.T399 is the second most predictive SNP. This allelle confers a threefold increased risk of fibrosis progression over carriers of the T399I variant, indicating a protective role in fibrosis progression of the c.1196C→T (rs4986791) variant at this location (p.T399I), along with another highly co-segregated SNP, c.896A→G (rs4986790), located at coding position 299 (p.D299G). Absence of TLR4 or expression of the TLR4 T399I and/or D299G SNPs confers reduced LPS responsiveness in cultured human or mouse HSCs [72]. These SNPs reduce NF-κB activation and pro-inflammatory cytokine expression, and attenuate downregulation of the TGF-β pseudoreceptor BAMBI in HSCs after LPS stimulation. These SNPs also reduce cell growth and lower the apoptotic threshold in mouse hepatic stellate cells after apoptotic stress [72]. In addition to these two missense variants, other variants of the TLR4 gene have also been independently associated with the risk of fibrosis by dense genotyping and association testing, findings that warrant further mechanistic studies [146]. Thus, although specific SNPs confer LPS hypo-responsiveness and increased susceptibility to infection, they reduce the likelihood of end-organ damage due to progressive scarring. Further studies are needed to explore if these SNPs also affect fibrogenesis through responses in other cell types and if they affect the response of TLR4 to endogenous ligands (Figure 3).

## Future prospects

Because TLR4 signaling has been identified as a key inflammatory and fibrogenic signal in injured liver and HSCs, interventions to inhibit the intracellular signaling associated with TLR4-IL-1R might be effective in reducing the inflammatory actions of TLR4-mediated liver injury and dampening liver fibrogenesis [147]. A peptide termed P13 limits the LPS-induced inflammatory response and enhances survival in murine models of inflammation [148]. Pharmacological inhibition of endotoxin responses has also been achieved by targeting the TLR4 co-receptor, MD2 [149]. Several small molecule inhibitors of TLR4 are currently being tested, including:

(1) Lipid A mimetics (for example, E5564 and CRX-526 [150,151]) which bind to the TLR4-MD2 complex but lack intrinsic activity and thus prevent binding of the lipid A portion of LPS and subsequent TLR4 activation

(2) TAK-242, which exerts its inhibitory effects at the intracellular domain of TLR4 [152]. Both E5564 and TAK-242 are currently being tested in phase III clinical trials in patients with septic shock.

(3) Soluble fusion proteins of the extracellular domain of TLR4 and MD2 or TLR4/MD2/IgG-Fc fusion protein that bind LPS. They specifically inhibit LPS-induced NF-κB and JNK activation, and abolish LPS-induced secretion of chemokines (MCP-1) and cytokines (IL-6) from HSCs [153]. This soluble receptor might provide a new biologic agent in the prevention and therapy of liver fibrosis and other diseases in which TLR4-mediated signal transduction plays a pathological role, such as in alcoholic liver injury and non-alcoholic steatohepatitis.

(4) Fc/fusion protein or antagonists of TREM-1 (triggering receptor expressed on myeloid cells-1), which belongs to another pattern recognition receptor family. This molecule synergizes with TLR4, and mediates the inflammatory responses of hepatic macrophages and endothelial cells to LPS. Blockage of TREM-1 limits LPS-induced inflammatory responses and injury [154].

By contrast, synthetic TLR4 agonists may boost the protective innate immune responses against infection [155]. Examples include alpha-1 acid glycoproteins, which are a class of lipid A mimetics composed of a monosaccharide unit with an N-acylated aminoalkyl aglycon spacer arm. Increasing evidence suggests that immune modulators such as TLR4 ligands or agonists could also be successfully used as therapeutic agents in infectious liver diseases, such as HBV and HCV [93,156].

## Competing interests

The authors declare that they have no competing interests.

## Authors' contributions

JG wrote the initial draft of the manuscript and figures. SLF finalized the manuscript and provided guidance into its organization and figures. All authors read and approved the final manuscript.
